# Disruption of Host-Symbiont Associations for the Symbiotic Control and Management of Pentatomid Agricultural Pests—A Review

**DOI:** 10.3389/fmicb.2020.547031

**Published:** 2020-11-27

**Authors:** Elena Gonella, Bianca Orrù, Ramona Marasco, Daniele Daffonchio, Alberto Alma

**Affiliations:** ^1^Dipartimento di Scienze Agrarie, Forestali e Alimentari, Università degli Studi di Torino, Turin, Italy; ^2^Biological and Environmental Sciences and Engineering Division, Red Sea Research Center, King Abdullah University of Science and Technology, Thuwal, Saudi Arabia

**Keywords:** stink bug, gut symbiont, *Pantoea*, vertical transmission, egg smearing, pest control

## Abstract

The family Pentatomidae (Hemiptera: Heteroptera) includes several invasive stink bug species capable to attack a large number of wild and cultivated plants, causing several damages to different crops. Pentatomids rely on obligate symbiotic associations with bacteria of the family *Enterobacteriaceae*, mainly of the genus *Pantoea*. A distinctive trait of these associations is the transmission route: during oviposition, females smear egg masses with symbiont-containing secretions, which are ingested by newly hatched nymphs, allowing the symbiont to pass through their digestive tract and establish in the crypts of the posterior midgut. Preventing newborns from orally acquiring symbionts seriously affects their fitness and survival. This symbiont inheritance process can be manipulated to develop innovative pest control measures by sterilization of egg masses prior to nymph hatching. This review summarizes the recent knowledge advances concerning the gut primary symbionts of pentatomids, with a specific focus on the most troubling pest species for agriculture. Current understanding of host colonization dynamics in pentatomids is presented, as well as the phenotypic effects determined in different insect species by the alteration of vertical transmission. Details on the current knowledge on the whole bacterial communities accompanying primary symbionts are analyzed. The recent research exploiting the perturbation of symbiont acquisition by pentatomid nymphs is discussed, by considering published work on laboratory and field trials with several active substances. These translational strategies are presently regarded as promising for limiting the populations of many important pentatomid pests in a sustainable way.

## Introduction

Obligate symbioses with bacteria are widespread in many insect orders, and have particular evolutionary significance in species with nutritionally restricted diets. A main function of obligate symbionts is nutrient supply, upgrading the biosynthetic properties of the hosts and consequently their feeding potential (Buchner, 1965; Jing et al., 2020). A crucial phase for insect-microbe obligate symbioses is symbiont inheritance, which is essential for the evolutionary conservation of symbiosis-related advantages (Salem et al., 2015). This process is allowed by transgenerational (vertical) transmission (i.e., the bacterial transfer from mother to offspring), which may take place in different ways according to the bacterial localization within the host body (Hurst, 2017). Intracellular symbionts can be transmitted from the mother to the offspring by entering the ovaries and the germline (i.e., transovarial transmission). One of the most widespread transovarially transmitted symbionts is the alphaproteobacterial *Wolbachia* that, by colonizing the egg cytoplasm, induces a number of manipulations of the host reproduction to the advantage of infected females and its own dissemination (Werren et al., 2008). Extracellular symbionts undergo alternative transmission strategies, including physical deposition of symbiont-containing substances close to the eggs after oviposition (e.g., egg smearing or capsule production), or environmental reacquisition of symbiotic cells by each generation (Hurst, 2017; Hosokawa and Fukatsu, 2020). Extracellular transmission routes imply that newborns are aposymbiotic after emergence, being exposed to possible failures of symbiont acquisition. The manipulation of symbiont vertical transmission in insect pests has been recently regarded as a promising method for implementing pest control strategies. Target insects for the application of such an approach must retain the following biological and behavioral traits: (i) nutritional dependence on bacterial symbiont; (ii) vertical transmission based on symbiont reacquisition by newborns; and (iii) well-delimited reacquisition sites. Insects characterized by transmission of associated symbionts through maternal secretions possess all of these features, therefore they are amenable to vertical transmission disruption.

Inherited symbiotic relationships are very common in the order Hemiptera. Associations are well-recognized in Auchenorrhyncha and Sternorrhyncha and, in the Heteroptera, they are usually found in infraorders Cimicomorpha and Pentatomomorpha (Hosokawa et al., 2006, 2019; Duron and Noël, 2016). In this review, a special focus is given to inherited symbionts of the Heteroptera, as they include many important pests in agriculture, such as the stink bugs (Pentatomidae; Knight and Gurr, 2007; Palumbo et al., 2016; Leskey and Nielsen, 2018; Sosa-Gómez et al., 2020), shield bugs (Scutelleridae; Davari and Parker, 2018), and plataspids (Plataspidae; Dhammi et al., 2016). More specifically, we describe host colonization and vertical transmission of gut symbionts of the family Pentatomidae. This family encompasses a number of agricultural pests that are responsible for huge economic losses worldwide, as they feed on a variety of fruits and seeds, seriously affecting crop yield and quality (Conti et al., 2020). Heavy attacks are recorded on almost all economically relevant crops, including commodity crops such as cotton, rice, maize, soybean, and wheat (Leskey and Nielsen, 2018; Zerbino and Panizzi, 2019; Defensor et al., 2020; Sosa-Gómez et al., 2020), fruit trees (Leskey and Nielsen, 2018; Mi et al., 2020; Powell, 2020), nuts (Bosco et al., 2018; Mehrnejad, 2020), and vegetables (Palumbo et al., 2016; Leskey and Nielsen, 2018). Furthermore, the invasive potential of many pentatomid species, such as *Halyomorpha halys* (Stål), *Nezara viridula* L., *Erthesina fullo* Thunberg, or *Bagrada hilaris* (Burmeister), makes them major agricultural threats in several areas outside their native range (Palumbo et al., 2016; Leskey and Nielsen, 2018; Conti et al., 2020; Mi et al., 2020).

Hemipteran species are associated with different bacterial taxa of Actinobacteria, *Alphaproteobacteria, Bacteroidetes, Betaproteobacteria, Gammaproteobacteria*, and Firmicutes (Sudakaran et al., 2017; Kashkouli et al., 2020). In Heteroptera, the ubiquitous inherited endosymbiont *Wolbachia* has been recorded in several species (Kikuchi and Fukatsu, 2003), even though it is not so widespread as in other insect taxa, and its role on the hosts biology has been poorly investigated (Kikuchi and Fukatsu, 2003; Matsuura et al., 2012; Becerra et al., 2015).

*Gammaproteobacteria* are one of the most represented taxa in the Heteroptera suborder hosts (Bansal et al., 2014; Karamipour et al., 2016; Kashkouli et al., 2020). These symbionts are essential for growth, development, and survival of the host and generally exhibit particular genomic features, such as A+T enriched genomes, fast-tracked molecular evolution and drastically reduced genome size to <1 Mb (Nikoh et al., 2011).

In Pentatomidae, bacteria of the family *Enterobacteriaceae* are often related to the genus *Pantoea* (Duron and Noël, 2016). These symbionts are obligate mutualists, provide their hosts with missing nutrients in their diets (Kenyon et al., 2015; Otero-Bravo et al., 2018), and inhabit crypts of the terminal portion of midgut, named region V4 (Fukatsu and Hosokawa, 2002; Prado et al., 2006; Karamipour et al., 2016). The maintenance of such primary symbionts in the host is ensured by egg smearing, i.e., deposition of maternal secretions upon egg surface, containing symbiont cells, which are orally acquired by newborns after hatching (Prado et al., 2006; Tada et al., 2011; Bansal et al., 2014; Otero-Bravo and Sabree, 2015). During the transmission process, the persistence of symbionts in the extrachorion matrix—outside the insect tissues—is supported by genomes that, even though reduced with respect to their free-living counterparts, are larger than those of intracellular primary symbionts commonly found in other Hemiptera. Their genomes retain genes encoding essential factors for autonomous life, such as those for the cell wall synthesis (Bergmann et al., 2014). These symbionts are adapted to multiple lifestyles, such as the symbiotic and the environmental lifestyles. Under an operational framework of sustainable pest control strategies, the environmental phase of the life cycle of these bacteria can be exploited to interfere with the symbiont acquisition process by the newborns that may impair their development (Taylor et al., 2017; Gonella et al., 2019; Kashkouli et al., 2019b).

## Symbiotic Associations in Pentatomids

A growing number of studies highlighted the importance of gut symbioses in stink bugs, with emphasis on their peculiar transmission routes. According to the behavioral traits regulating the inheritance of pentatomid gut symbionts, upon hatching, first-instar nymphs remain aggregated around the egg masses and orally ingest the symbionts laid on the eggs (**Figure 2**) (Kashkouli et al., 2019a; Oishi et al., 2019). During the host development the ingested symbionts colonize the inner cavities of the crypts in the posterior midgut of the second instar (Hosokawa et al., 2006; Oishi et al., 2019). Initial investigations of the adult digestive tract revealed that the symbiont-inhabited gut crypts are attached to the main gastric region by means of connective tissue, with no evident communication between these structures, suggesting that establishment in the crypts occur immediately after ingestion (Nikoh et al., 2011; Bansal et al., 2014). Indeed, a study of the spatiotemporal dynamics of the symbiont colonization process during the early developmental stages of the host showed the establishment of the gut bacteria in the posterior midgut already in the first instar (Oishi et al., 2019). Thickening and folding of the midgut epithelium proceed all throughout the immature development, with formation of several crypts and the spatial isolation of posterior midgut from the anterior part. In the final adult stage, bacteria are confined in the crypt cavities and isolated from the remaining gut compartments (Oishi et al., 2019). Morphological studies of the midgut of adult stink bugs highlighted a differentiation between males and females, with females displaying enlarged posterior midgut and bacteria occurring in the main tract outside of crypts, to allow egg smearing of symbiotic cells during oviposition (Hayashi et al., 2015).

**Figure 1:**
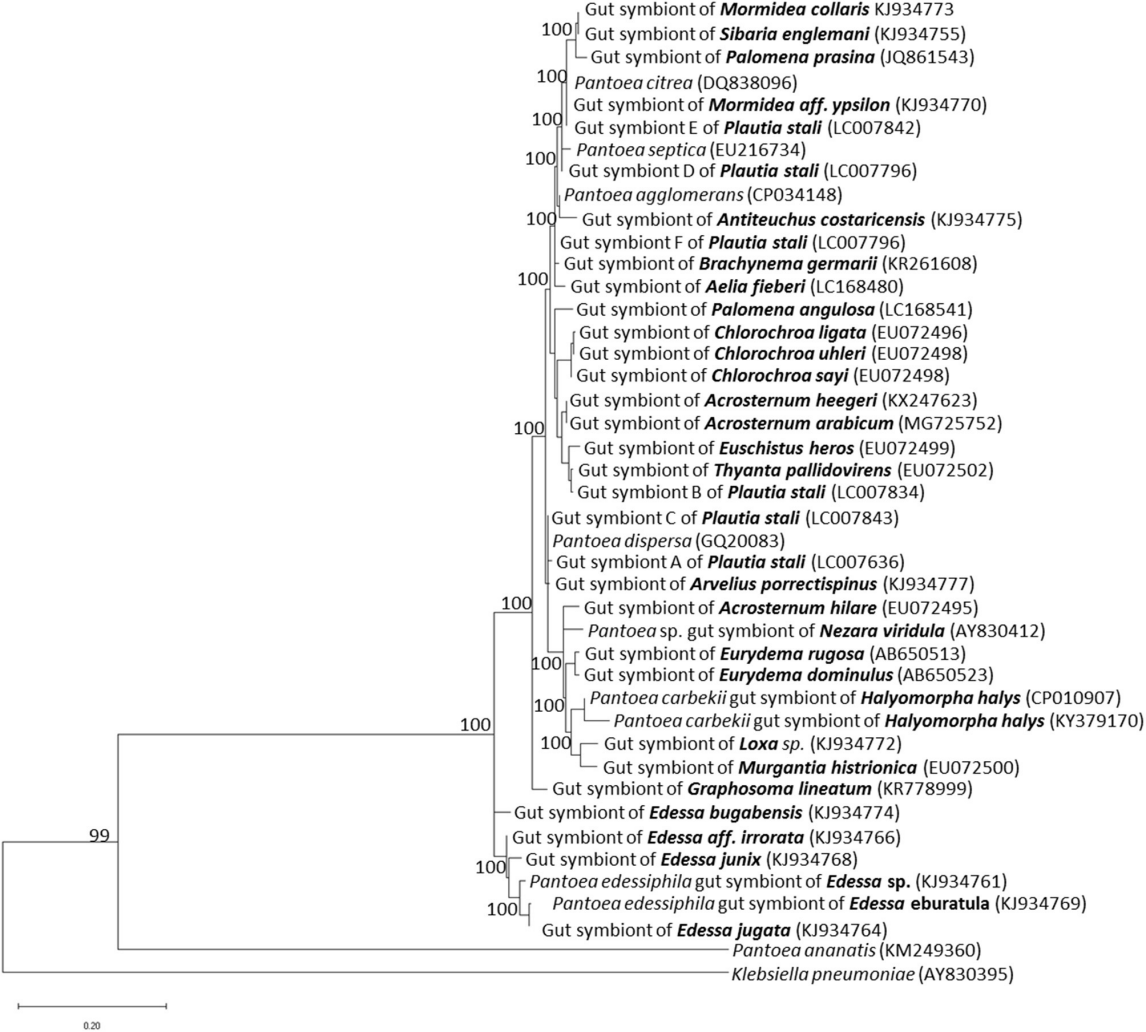
Phylogenetic placement of gut primary symbionts of pentatomid stink bugs. The phylogenetic tree was constructed by the maximum likelihood method, using the MEGA X software. The 16S rRNA gene sequences of pentatomid gut symbionts, available in public databases, were selected according to: Hirose et al. (2006), Prado and Almeida (2009a), Kikuchi et al. (2012), Kenyon et al. (2015), Bistolas et al. (2014), Hosokawa et al. (2016), Karamipour et al. (2016), Otero-Bravo and Sabree (2018), and Kashkouli et al. (2019a). Allied *Pantoea* species are included. Names of pentatomid hosts are indicated in bold; accession numbers of each sequence included in the analysis are indicated in parenthesis. Numbers at each node represent percentages of bootstrap replications calculated from 1,000 replicate trees. The scale bar represents the sequence divergence. *K. pneumoniae* was used as an outgroup in the family *Enterobacteriaceae*.

Molecular and genomic methods identified through sequences of the 16S rRNA gene the primary symbiont of different stink bugs (Bansal et al., 2014), which were mostly classified within the genus *Pantoea*, closely related to plant- and insect-related strains (Prado and Almeida, 2009a) (Figure 1). Phylogenetic analyses of pentatomid symbionts indicated that they are polyphyletic, suggesting that several events of taxonomically-related symbiont replacements took place among different hosts during evolution. Evidences of different levels of dependency on symbiotic relationships have been provided for distinct stink bug species (Prado and Almeida, 2009a).

A considerable number of pentatomid primary symbionts and their importance for the host life cycle completion have been described, and a number of aberrant biological traits have been identified in response to a missed symbiont acquisition (Table 1). Some of the currently described host-symbiont systems, which have been studied in detail due to the major economic relevance of the stink bug host, are examined below.

**Table 1:** List of described gut primary symbionts of pentatomid hosts.

**Host insect**	**Primary symbiont**	**Symbiont suppression modality**	**Symptoms of symbiont suppression**	**Symbiont-targeted management**	**References**
*A. arabicum*	Unnamed	Heat treatment; surface sterilization (ethanol + bleach)	>Nymphal mortality; <Adult fecundity	Laboratory trials	Kashkouli et al., 2019b
*A. heegeri*	*Pantoea* sp.	Heat treatment; surface sterilization (ethanol + bleach)	>Pre-adult development; <Adult longevity; < Adult fecundity	Laboratory trials	Kashkouli et al., 2019a,b
*Acrosternum hilare*	Unnamed	Surface sterilization (ethanol + bleach)	> Developmental time; >Nymphal mortality; < Adult fecundity	N. a.	Prado and Almeida, 2009a,b
*Antiteuchus costaricensis*	Unnamed	N. d.	N. d.	N.a.	Bistolas et al., 2014
*Arvelius porrectispinus*	Unnamed	N. d.	N. d.	N. a.	Bistolas et al., 2014
*B. germari*	*Pantoea* sp.	Heat treatment; surface sterilization (ethanol + bleach)	>Pre-adult development; < Adult longevity; < Adult fecundity	Laboratory trials	Kashkouli et al., 2019a,b
*Chlorochroa ligata*	Unnamed	N. d.	N. d.	N. a.	Prado and Almeida, 2009a
*Chlorochroa sayi*	Unnamed	N. d.	N. d.	N. a.	Prado and Almeida, 2009a
*Chlorochroa uhleri*	Unnamed	N. d.	N. d.	N. a.	Prado and Almeida, 2009a
*Edessa bella*	“*Ca*. Pantoea edessiphila”	N. d.	N. d.	N. a.	Bistolas et al., 2014; Otero-Bravo et al., 2018
*Edessa bugabensis*	Unnamed	N. d.	N. d.	N. a.	Bistolas et al., 2014
*Edessa eburatula*	“*Ca*. Pantoea edessiphila”	N. d.	N. d.	N. a.	Bistolas et al., 2014; Otero-Bravo et al., 2018
*Edessa aff. irrorata*	Unnamed	N. d.	N. d.	N. a.	Bistolas et al., 2014
*Edessa jugata*	Unnamed	N. d.	N. d.	N. a.	Bistolas et al., 2014
*Edessa junix*	Unnamed	N. d.	N. d.	N. a.	Bistolas et al., 2014
*Edessa loxdalii*	“*Ca*. Pantoea edessiphila”	N. d.	N. d.	N. a.	Otero-Bravo et al., 2018
*Edessa n*. sp.	“*Ca*. Pantoea edessiphila”	N. d.	N. d.	N. a.	Otero-Bravo et al., 2018
*Eurydema dominulus*	Unnamed	N. d.	N. d.	N. a.	Kikuchi et al., 2012
*Eurydema rugosa*	Unnamed	Surface sterilization (ethanol + formaldehyde)	Retarded growth; < Body weight; Abnormal body color	N. a.	Kikuchi et al., 2012
*Euschistus heros*	Unnamed	N. d.	N. d.	N. a.	Prado and Almeida, 2009a
*Euschistus* sp.	Unnamed	N. d.	N. d.	N. a.	Bistolas et al., 2014
*Graphosoma lineatum*	Unnamed	Surface sterilization (ethanol + bleach)	>Developmental time; < Lifespan; < Adult fecundity	N. a.	Karamipour et al., 2016
*H. halys*	“*Ca*. Pantoea carbekiii”	Surface sterilization (bleach); treatments with several antimicrobials and surfactants	>Nymphal mortality; >Developmental time; <Adult fecundity; <Progeny survivorship	Laboratory and field trials	Mathews and Barry, 2014; Taylor et al., 2014, 2017; Gonella et al., 2019
*Loxa* sp.	Unnamed	N. d.	N. d.	N. a.	Bistolas et al., 2014
*Mormidea collaris*	Unnamed	N. d.	N. d.	N. a.	Bistolas et al., 2014
*Mormidea aff. ypsilon*	Unnamed	N. d.	N. d.	N. a.	Bistolas et al., 2014
*Murgantia histrionica*	Unnamed	Surface sterilization (ethanol + bleach)	>Developmental time	N. a.	Prado and Almeida, 2009a,b
*N. viridula*	*Pantoea* sp.	Surface sterilization (ethanol + bleach; ethanol + formaldehyde); heat treatment	=Fitness >Nymphal mortality; Retarded growth; <Size; Abnormal body color	N. a.	Prado et al., 2006; Tada et al., 2011; Kikuchi et al., 2016
*P. guildinii*	Unnamed	N. d.	N. d.	N. a.	Husseneder et al., 2017
*P. stali*	*Pantoea* spp.	Surface sterilization (ethanol + formaldehyde)	>Nymphal mortality	N. a.	Abe et al., 1995; Hosokawa et al., 2016
*Sibaria englemani*	Unnamed	Surface sterilization (bleach)	>Duration of II instar; <Growth rate; Aberrant gut morphology	N. a.	Bistolas et al., 2014
*Thyanta pallidovirens*	Unnamed	N. d.	N.d.	N. a.	Prado and Almeida, 2009a

*The modalities tested for symbiont suppression and the resulting effects are indicated, as well as the current advancement of development of pest management strategies, when available. N. d., not determined; N. a., not available; =, no significant changes observed; >, significant increment; <, significant reduction; Ca., Candidatus*.

### Halyomorpha halys

The brown marmorated stink bug *H. halys* is an invasive Asiatic pentatomid recently introduced in North America and Europe. Its marked polyphagy—it attacks more than 300 species of plants—, high reproduction potential and high mobility make this insect a major pest of many crops (Rice et al., 2014; Leskey and Nielsen, 2018). The gut primary symbiont of *H. halys* is “*Candidatus* Pantoea carbekii” (*P. carbekii*) (Bansal et al., 2014), one of the few stink bug symbiotic bacteria whose genome is currently available (Kenyon et al., 2015). *P. carbekii* genome analysis, besides showing the genetic potential for nutrient provisioning to the host, indicated that this bacterium shares some genomic traits with intracellular primary symbionts of insects, such as a reduced genome size (0.7–0.9 Mb) and a low G+C content (Kenyon et al., 2015). These are both distinctive features of endosymbionts with stable host-restricted lifestyles (Moran et al., 2008). However, *P. carbekii* still encodes functional genes for essential extracellular life style traits, such as the metabolic pathways for the peptidoglycan synthesis, the generation of ATP by aerobic respiration, and other primary metabolic processes (Kenyon et al., 2015).

Prevention of symbiont acquisition through surface-sterilization of *H. halys* eggs results in nymph developmental delays in the first generation, providing direct evidence of its high dependence on *P. carbekii* (Taylor et al., 2014). Furthermore, in the second generation, only few individuals reach the adult stage, and surviving adults show longer pre-oviposition periods and produced less eggs, which in turn drastically reduces hatching rates and juvenile survivorship (Taylor et al., 2014).

### Nezara viridula

The southern green stinkbug *N. viridula* is a cosmopolitan species distributed in different regions of North and South America, Africa, Asia, Australia, and Europe, and known as an important agricultural pest that damages a large number of crop plants (Tada et al., 2011). An unnamed symbiotic bacterium allied to the *Enterobacteriaceae* was reported as the gut symbiont of this stink bug (Hirose et al., 2006; Prado et al., 2006). Its involvement in *N. viridula* life cycle is debated, since different stink bug populations resulted either affected or not by symbiont removal (Prado et al., 2006; Tada et al., 2011). In facts, studies on Hawaian populations showed no clear fitness decrease on the offspring emerging from surface-sterilized egg masses, as emerged nymphs reached adulthood, finally producing viable eggs, despite they were symbiont-free (Prado et al., 2006). In contrast, similar treatment on egg masses from a Japanese population resulted in high mortality, with only few individuals reaching the adult stage (Tada et al., 2011). In this population, heat-induced symbiont suppression induced several fitness abnormalities, including retarded growth, reduced size, and altered body color (Kikuchi et al., 2016).

### Plautia stali

The brown-winged green stinkbug *Plautia stali* Scott is a harmful pest of several fruit trees and crops (Oishi et al., 2019). *P. stali* was firstly reported to be associated with a single specific gammaproteobacterial symbiont, allied to *Pantoea* sp. This is an uncultivable bacterium with a small genome and it is essential for normal growth, fecundity, and survival of the insect host (Hosokawa et al., 2008; Oishi et al., 2019). Interruption of symbiont vertical transmission seriously hampers the development of nymphs hatched from sterilized eggs, due to the arise of opportunistic infections (Abe et al., 1995). Genomic analysis of the *P. stali* symbiont suggests that it is capable of producing lipopolysaccharides (LPS), important antigenic components of the Gram-negative bacteria cell wall, which are likely exploited as a defense during the environmental lifestyle, and as trigger for innate immune response of the host against pathogens (Kobayashi et al., 2011).

*P. stali* and its associated symbiont are currently regarded as a laboratory model for studying the insect-microbe gut symbiosis (Oishi et al., 2019). Phylogenetic investigations on *P. stali* symbionts showed six distinct bacterial linages, differing for their genome size, cultivability and prevalence in the host populations. All the six lineages were demonstrated to be individually essential for the host survival: after the elimination of the original symbionts, cross colonization of the host by alternative lineages completely restored insect development and growth, suggesting the possibility of multiple replacements. Notably, aposymbiotic nymphs were able to acquire symbionts from the environment (for example from the food source), suggesting also a possible horizontal route for symbiont replacements in nature (Hosokawa et al., 2016; Nishide et al., 2017).

### Pistachio Green Stink Bugs

Pentatomid stink bugs are abundant and serious pests of pistachio nuts in most of pistachio plantation areas. They are reported to cause heavy losses in Iran due both to direct damage by the insect and the transmission of the fungal pathogen, *Nematospora coryli* Peglion, a pathogen of pistachio nuts (Pourkhatoon et al., 2016). This group of stink bugs includes the species *Acrosternum arabicum* Wagner, *Acrosternum heegeri* Fieber, *Acrosternum millieri* Mulsant & Rey, *Apodiphus amygdali* Germar, *Brachynema germari* Kolenati, and *Brachynema segetum* Jakovlev (Kashkouli et al., 2019a; Mehrnejad, 2020). Most of information concerning the associations with gut primary symbionts regards *B. germari* and *Acrosternum* spp., in consideration of their primary importance as pests (Kashkouli et al., 2019a). In *B. germari*, a peculiar bacterial distribution was observed in the V4 midgut region, with the obligate symbiont sheltered in the intercellular space rather than intracellular cytoplasm (Hosokawa et al., 2006). Molecular phylogenetic analyses of 16S rRNA gene of *B. germari* and *A. heegeri* symbionts placed these bacteria in the genus *Pantoea* (Kashkouli et al., 2019a, 2020). A comprehensive study of the effects on hatched nymphs of these species after surface sterilization or heat treatment of egg masses showed high nymph mortality, slow growth, reduced fitness and reduced fecundity (Karamipour et al., 2016; Kashkouli et al., 2020).

## Obligate Symbionts as Part of Pentatomid Microbiomes

Compared to the relatively large body of information on the stink bug obligate symbionts, few data are available concerning the other members of the gut microbial community and their interactions with the main symbionts. Studies of the gut microbiome have been conducted on adult individuals of few pentatomid species, like *H. halys* and *N. viridula*, revealing that the bacterial community of the crypt-harboring midgut portion (V4) is largely dominated by the primary symbionts (Kenyon et al., 2015; Medina et al., 2018), but yet colonized by other bacterial phyla in many stink bug hosts. For instance, in a survey on seven pentatomid species, different Actinobacteria were detected in the terminal midgut portion, mostly in the genera *Corynebacterium, Dietzia, Citricoccus, Mycobacterium, Propionibacterium*, and *Streptomyces*. These bacteria are thought to be involved in the protection of the microbial community, by producing bioactive metabolites which may limit the invasion of pathogens (Zucchi et al., 2012).

In the other midgut compartments (named V1-V3), the primary symbionts are much less abundant (Medina et al., 2018), and many other bacteria have been found. For example, in the digestive tract of the red-banded stink bug *Piezodorus guildinii* (Westwood) several bacteria putatively involved in nutrient provision and digestion have been identified, such as *Klebsiella oxytoca, Clostridium butyricum*, and *Citrobacter farmeri*, along with the candidate primary symbiont *Pantoea dispersa* (Husseneder et al., 2017). The analysis of different *N. viridula* populations from Brazil, Hawaii, California and Japan, confirmed that the terminal midgut ventriculus was dominated by a single bacterial type of the family *Enterobacteraceae*, while in the remaining compartments of the gut other Enterobacteria and Enterococci were detected (e.g., *Klebsiella pneumoniae, Enterococcus faecalis*, and *Yokenella* sp.), possibly being involved in detoxification of the food source (Medina et al., 2018). Many stink bugs are recognized as vectors of different plant pathogens, which often colonize the digestive tract, representing an additional component of the gut microbiota (Mitchell, 2004; Esquivel et al., 2010; Esquivel and Medrano, 2014; Medrano et al., 2016).

Even though a number of reports have described a relatively complex microbial community accompanying the primary symbionts of pentatomid bugs, at present no studies tackled the questions related to their ecological role and the possible interactions among these microorganisms and the primary symbionts to maintain the host fitness, or the mechanisms ruling microbial compartmentalization in the distinct gut portions. Future studies on the machinery of symbiotic homeostasis and microbiome dynamics will allow a better comprehension of the phenotypic effects observed in stink bugs in response to disruption of the association with the obligate symbionts.

## Disruption of Symbiont Inheritance for Pest Control Applications

**Figure 2:**
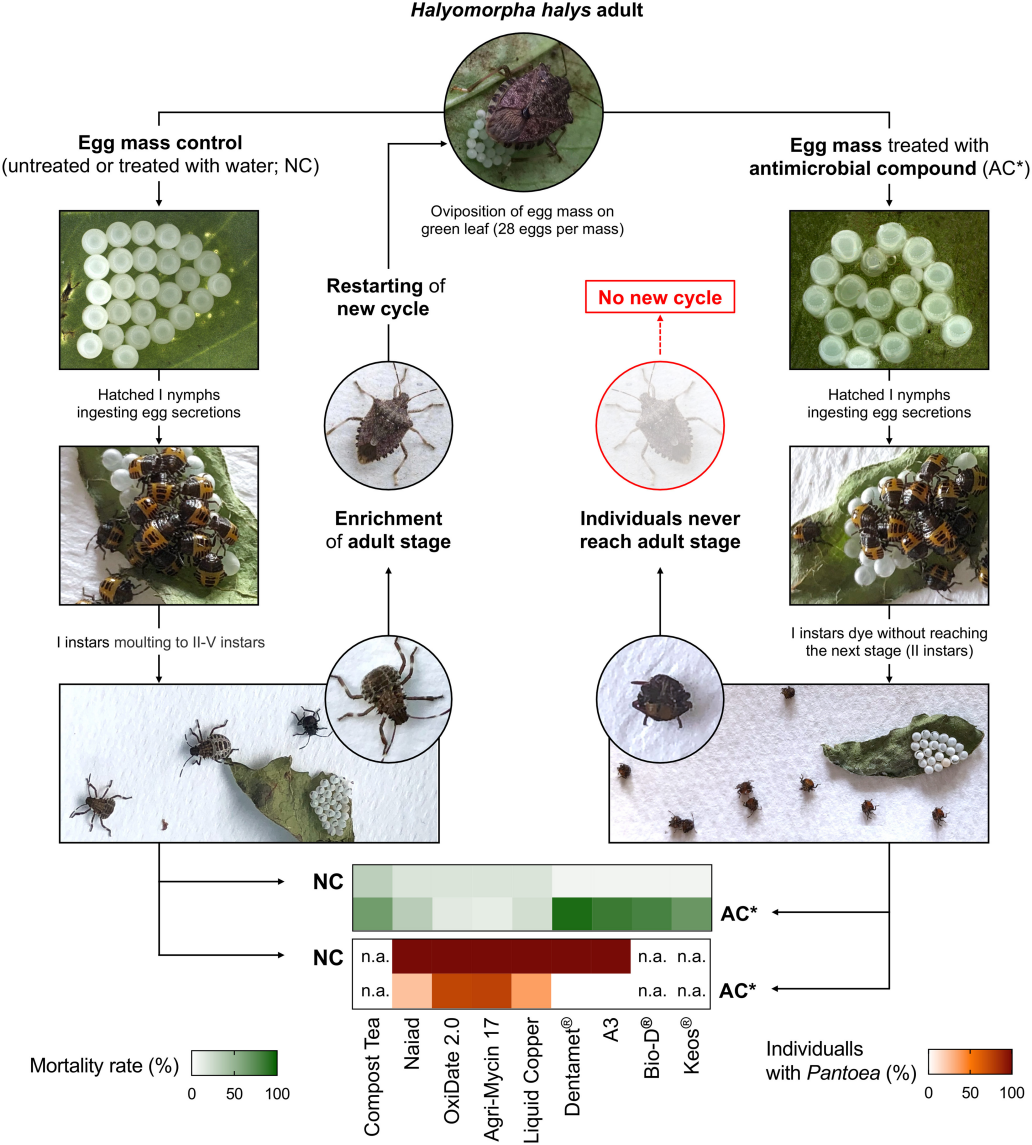
Graphical representation of the laboratory trails testing the anti-*P. carbekii* activity of commercial products with antimicrobial activity, interrupting the life cycle of *H. halys* (Mathews and Barry, 2014; Taylor et al., 2017; Gonella et al., 2019). The effect of treating with antimicrobial compounds (AC) is compared with a negative control (NC), which shows no alteration of stink bug development. Mortality rates induced by different substances, as well as P. *carbekii* infection rates related to treatments, are depicted in the heatmaps.

Based on scientific evidences showing the harmful effect of preventing symbiont acquisition by nymphs through surface-sterilization of stink bug eggs (Abe et al., 1995; Tada et al., 2011; Taylor et al., 2014; Kashkouli et al., 2020), the creation of symbiotic control programs was envisaged against some pentatomid pests of major economic relevance (Table 1). For example, the use of surface-sterilizing agents was proposed as management tactic against pistachio green stink bugs in Iran (Kashkouli et al., 2019b). However, most of the studies on symbiotic control programs were conducted on *H. halys* (Figure 2), which is one of the most damaging stink bug agricultural pests in North America and Europe, due to its high polyphagy and invasive potential (Leskey and Nielsen, 2018). The first substances tested to suppress the primary symbiont *P. carbekii* were compost teas, i.e., biologically-active organic matter emulsions, commonly used for pathogen management in organic agriculture (Mathews and Barry, 2014). The application of different compost teas, deriving from poultry manure and mushroom waste, resulted in high nymphal mortality in the first and second instar, especially when egg masses were treated few days after deposition (Mathews and Barry, 2014). These authors suggested that the observed reduction of insect survival could be related to antagonistic effects exerted by the complex microbiota hosted by the compost teas. However, besides a putative anti-*P. carbekii* activity, a direct insecticidal effect was reported against *H. halys* eggs, resulting in reduced egg hatching (Mathews and Barry, 2014).

A vast array of substances was tested by Taylor et al. (2017) on *H. halys* egg masses for their effects on the fitness of newly hatched nymphs. A laboratory assay was conducted with available products for plant pathogen control in the USA, including surfactants, insecticides, and different antimicrobials. The application of antimicrobial and surfactant formulates caused high nymphal mortality related to missed symbiont acquisition, providing evidence of the potential use of these products for the management of *H. halys*. However, the same substances only affected egg hatch rate in field experiments, showing no reduction of symbiont acquisition and nymphal survival (Taylor et al., 2017), and indicating the need to optimize control strategies (e.g., by identifying the most efficient number of treatments and doses) before proposing symbiont-targeted control options.

A different set of active substances, commercially available in the European agriculture as micronutrient fertilizers, were used against *H. halys* through primary symbiont elimination (Gonella et al., 2019). Egg mass treatments with a zinc, copper and citric acid biocomplex under laboratory conditions removed *P. carbekii*, as confirmed by molecular diagnosis, resulting in high mortality rates of the I instar nymphs. The suppressive effect was attributed to the anti-microbial activity of the Zn- and Cu-hydracid complexes contained in these micronutrient fertilizers, whose components are well-known antibacterial agents used for the control of plant pathogens (Gonella et al., 2019). However, these results still need to be experimentally validated under field conditions.

Notably, a significant negative relationships between the mortality rate and *P. carbekii* infection rate is highlighted by examining the results of laboratory tests conducted on *H. halys* egg masses by Taylor et al. (2017) and Gonella et al. (2019) using substances with an antimicrobial activity, suggesting commonalities in the nymph suppression processes exerted by different compounds that target the symbiont. The significant suppressive effect reported for several products, and the clear correlation observed between mortality rates and percentage of individuals carrying *P. carbekii*, further underline the importance to design symbiont-targeted control strategies against *H. halys*.

## Concluding Remarks

The family Pentatomidae includes many cosmopolitan and invasive species capable of large infestations, causing intense damage to several crops (Knight and Gurr, 2007; Palumbo et al., 2016; Leskey and Nielsen, 2018; Sosa-Gómez et al., 2020). Invasions in new areas may be very difficult to contain due to the limited knowledge of the pest life cycle in different new environments joint to the absence of effective natural enemies (McLaughlin and Dearden, 2019). Additionally, their actual control mainly relies on the use of chemical insecticides, which often determine hidden costs due to the environmental impact and the effect on human health. Another major management measure against pentatomid pests is biological control, taking advantage of specialized egg parasitoids (Correaferreira and Moscardi, 1995; Felipe-Victoriano et al., 2019; Moraglio et al., 2019; Sabbatini Peverieri et al., 2019). Their activity is seriously hampered by intensive use of chemicals (Lowenstein et al., 2019), leading to the need for sustainable alternatives (Brzozowski and Mazourek, 2018). The peculiar inheritance mode of bacterial symbionts of Pentatomidae is certainly an interesting target for the disruption of the stink bugs life cycle. Some of the active substances tested to interrupt symbiont acquisition by neonate stink bugs are already commercially available as antimicrobials or fertilizers, and the exploitation of their accessory effect against *H. halys* represents an actual sustainable control option, since they are allowed in organic farming (Mathews and Barry, 2014; Gonella et al., 2019). Because these products are not insecticides, they are not expected to determine unintended effects on non-target insects, including egg parasitoids, even though no studies have been published regarding the consequences of treatments with such products on non-target species. However, it must be pointed out that some active substances proposed for the interruption of symbiont vertical transmission displayed an ovicidal effect (e.g., compost teas), and this may indirectly hamper the activity of egg parasitoids. Moreover, in some countries, regulatory issues may arise concerning the use of micronutrient fertilizers, as their use for crop protection purposes has not been regulated yet.

Additionally, future studies could implement current knowledge on potential different mechanisms of antagonistic action, exploring different formulates that are used against microbial pathogens of many crops. For example, a promising source of anti-bacterial activity against pentatomid primary symbionts is represented by microbial biocontrol agents. Indeed, several strains are the base of commercial products that are currently used against plant pathogens (Ab Rahman et al., 2018), and studies on the effects on stink bug egg masses played by this class of substances may constitute a further step toward the implementation of effective pest control methods with low environmental impact.

Finally, attention is deserved by the observations on symbiont alteration in stink bugs exposed to increased temperatures as a result of climate change (Kikuchi et al., 2016). This is certainly a major issue to be addressed within the set-up of risk maps, an important component of Integrated Pest Management measures. Indeed, temperature increase may represent a perturbation for symbiont acquisition and development similarly to the direct bacterial elimination from the egg surface, seriously affecting prediction models of distribution range in areas of new pest introduction (Kikuchi et al., 2016), with a practical relevance for stink bug management.

## Author Contributions

EG, BO, and RM conceived, designed, and wrote the manuscript. BO and RM prepared the table. EG and RM prepared the figures. AA and DD critically reviewed the manuscript and contributed to its improvement. All authors approved the final version of the manuscript.

## Conflict of Interest

The authors declare that the research was conducted in the absence of any commercial or financial relationships that could be construed as a potential conflict of interest.
